# Hyperthyroidism and Malignancy: An Underrecognized Association

**DOI:** 10.7759/cureus.88104

**Published:** 2025-07-16

**Authors:** Arunima Das, Jesu Pencilin Yesuvadiyan, Karthikeyan Selvaraj, Subramani Balakrishnan, Sasikumar Pattabi

**Affiliations:** 1 General Surgery, Sree Balaji Medical College and Hospital, Chennai, IND; 2 Surgery, Sree Balaji Medical College and Hospital, Bharath Institute of Higher Education and Research, Chennai, IND

**Keywords:** follicular cell carcinoma, hurthle cell carcinoma, hyperthyroidism, thyroidectomy, thyroid storm

## Abstract

Thyroidectomy is the definitive treatment for malignant thyroid swelling, and its role in patients with concurrent hyperthyroidism is crucial for addressing both endocrine dysfunction and malignancy. This study reports the incidence of thyroid malignancy in a patient with persistent hyperthyroidism. We analyze postoperative outcomes, including recurrence rates and the management of thyroid hormone levels. Carcinoma thyroid is rare in hyperthyroidism, Hürthle cell carcinoma is a still rarer variant of thyroid cancer, and has been occasionally reported in association with hyperthyroidism.

This is a case of a 55-year-old hyperthyroid female patient presenting with a swelling in the neck for two years. This article explains the coexistence of a silent carcinoma in a hyperthyroid patient. We detail the diagnostic workup, surgical approach (extent of thyroidectomy and lymph node dissection), and postoperative management, specifically addressing the resolution of hyperthyroidism and the oncological outcomes in this complex patient population.

## Introduction

Hyperthyroidism is a condition where the thyroid gland produces excessive thyroid hormones, most commonly due to Graves’ disease or toxic nodules. Symptoms range from mild (tremors, insomnia, anxiety) to severe (palpitations, weight loss, and thyroid storm) [1]. This hormone excess leads to thyrotoxicosis, affecting multiple organ systems. Normally, T3 and T4 are regulated by thyroid-stimulating hormone (TSH), which is produced by the pituitary. In primary hyperthyroidism, the thyroid overproduces hormones despite low TSH, while in secondary hyperthyroidism, elevated TSH causes increased hormone production.

Thyroid carcinoma, a neoplasm of the thyroid epithelium, is the most common cancer of the endocrine system [2]. Thyroid carcinomas originate from different cells within the thyroid: papillary carcinomas (80% of cases), follicular carcinomas (14%, including the 3% Hürthle cell subtype), and anaplastic or undifferentiated carcinomas (2%) arise from follicular cells, while medullary carcinoma (4%) develops from medullary cells [2].

While thyroid carcinoma was initially believed to be rare in patients with hyperthyroidism, current theories on carcinogenesis suggest that thyroid-stimulating antibodies may play a role [3]. These antibodies can stimulate both thyroid growth and processes that promote invasion and blood vessel formation [4], as well as activate insulin-like growth factor pathways [3]. However, the American Thyroid Association states that thyroid cancer occurs in Graves’ disease in 2% or fewer cases [5].

Thyroid nodules, found on ultrasound in 30-70% of individuals, can be associated with thyroid carcinoma and are clinically significant because 4-6% of biopsied nodules are malignant [6]. Both the American and British Thyroid Associations recommend that all ultrasound-detected thyroid nodules be evaluated with fine needle aspiration in patients who have a medical history that indicates a high risk for thyroid carcinoma [7].

Thyroidectomy is the oldest and often the preferred treatment option for Graves' disease. Although it remains underutilized as a definitive therapy for hyperthyroidism, a recent large retrospective study has shown that thyroidectomy is just as effective as antithyroid drugs (ATDs) and radioiodine in restoring normal thyroid hormone levels within six weeks of treatment.

## Case presentation

**Figure 1:**
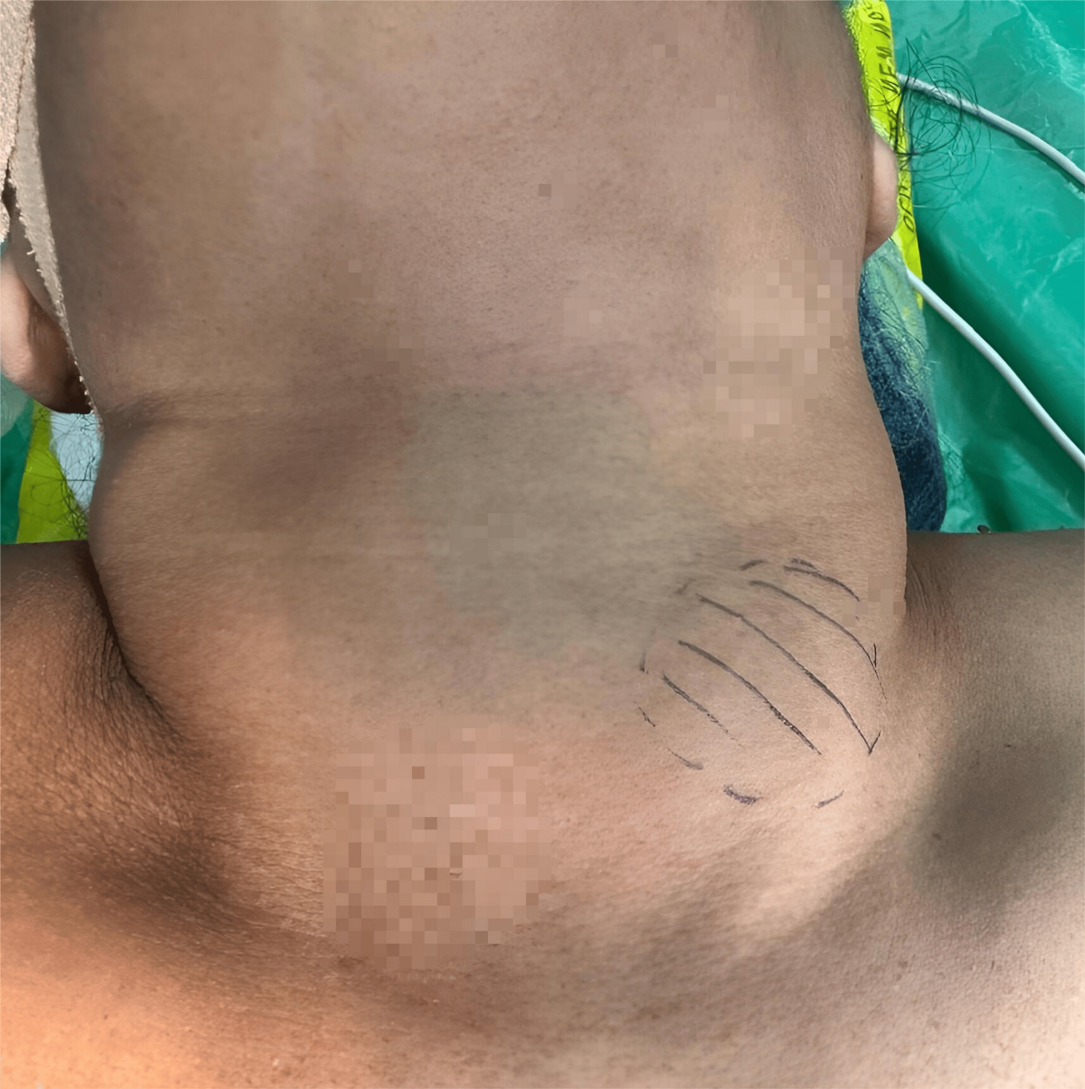
Preoperative image of the thyroid swelling.

A 55-year-old female presented to the surgery outpatient department as she noticed a swelling in front of the neck for the past two years, which was insidious in onset and gradually progressed to attain the current size of 3×4 cm. She also reported weight loss, insomnia, hair loss, and one episode of syncope (six months back). She had a history of tremors two years ago, which subsided after taking thyroid medication. She is a known case of hyperthyroidism for two years and has been on regular medication (tab. carbimazole). On examination, a swelling of 3x4 cm was present over the anterior aspect of the neck, which moves with deglutition (Figure 1). Swelling was extending superiorly to the thyroid cartilage, inferiorly 1 cm above the sternal notch, and laterally 2 cm from the sternocleidomastoid on either side.

Her thyroid function tests revealed that thyroid-stimulating hormone (TSH) was significantly suppressed at 0.01 mIU/L (reference range: 0.5-8.9 mIU/L), with elevated free T3 and free T4 levels (Table 1). Ultrasound of the neck consisted of a well-circumscribed hyperechoic nodule with few cystic spaces and a surrounding hypoechoic halo in the left lobe of the thyroid (Thyroid Imaging Reporting and Data System 3 {TIRADS 3}). Bilateral lobes of the thyroid show heterogeneous echotexture and increased vascularity.

**Table 1:** Initial thyroid function test (TFT) report.

Laboratory investigations	Patient's value	Institutional value
Thyroid-stimulating hormone	0.01 mIU/L	0.5-8.9 mIU/L
Free T3	4.27 ng/dL	2.1-4.4 ng/dL
Free T4	2.49 ng/dL	0.8-2.7 ng/dL
Ionized calcium	1.14 mmol/L	1.16-1.31 mmol/L

**Figure 2:**
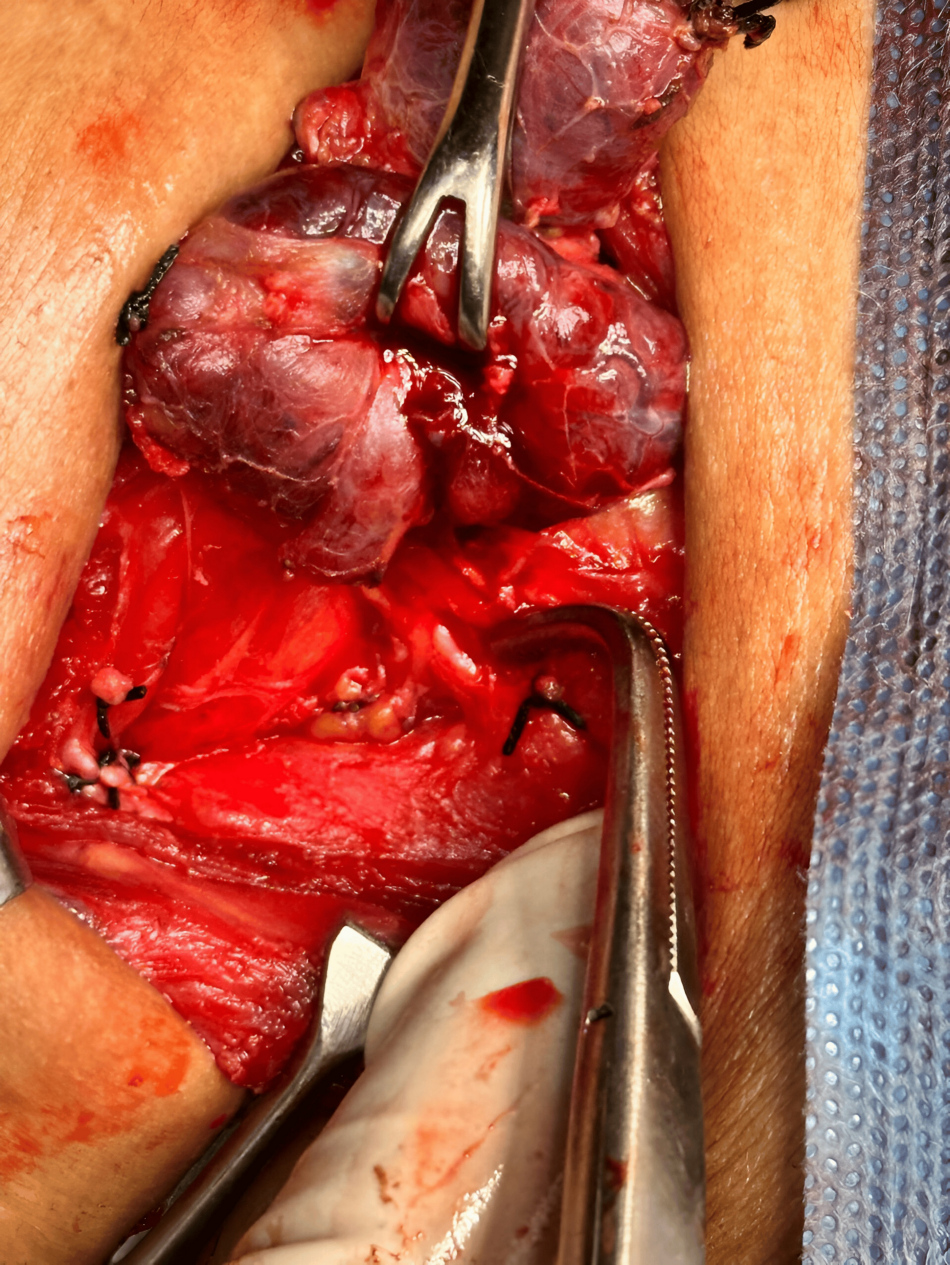
Intraoperative image of total thyroidectomy.

**Figure 3:**
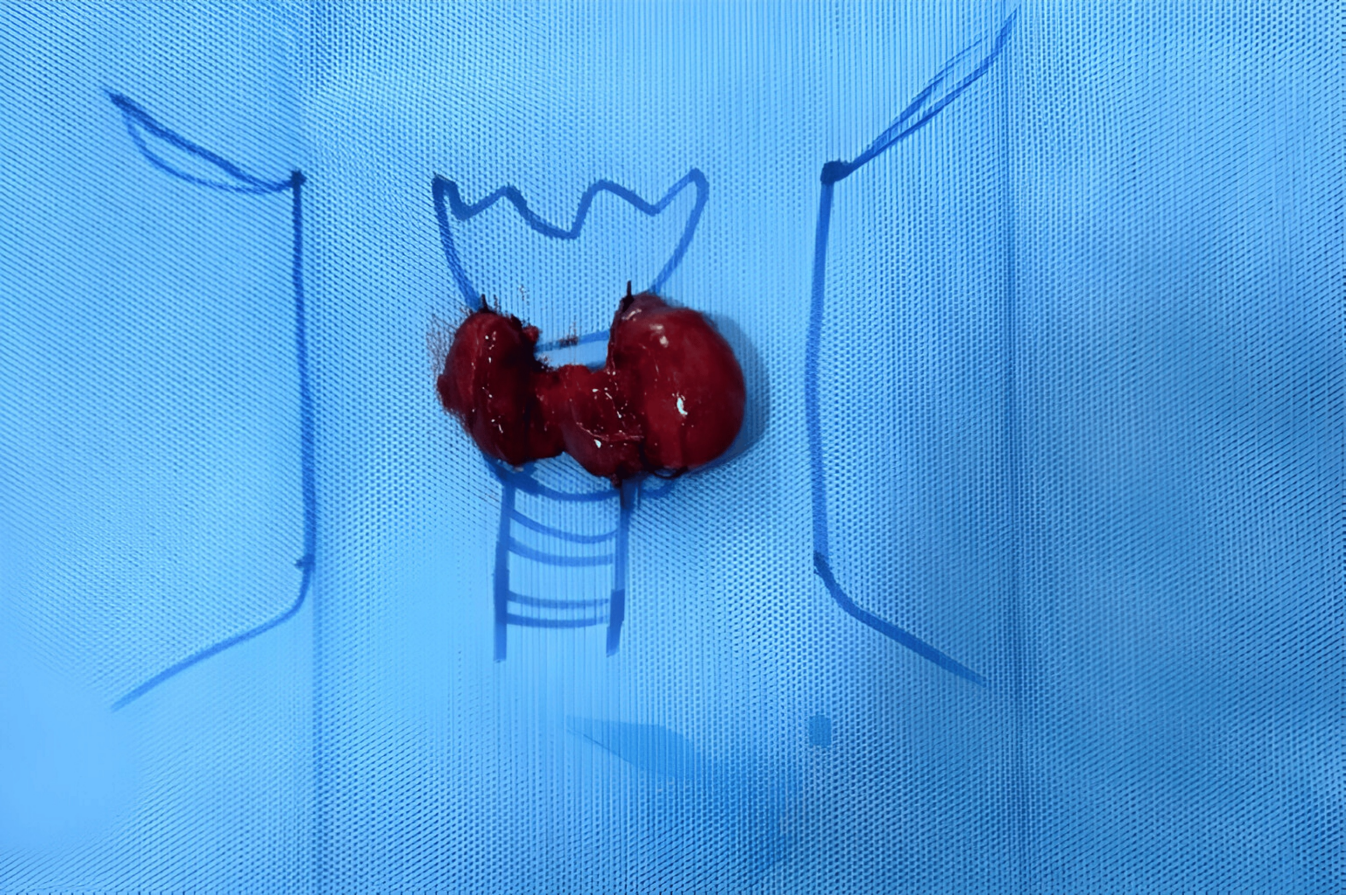
Post thyroidectomy specimen image.

Ultrasound-guided fine needle aspiration cytology (FNAC) revealed follicular nodular disease, nodular goitre with evolving thyroiditis (Bethesda System for Reporting Thyroid Cytopathology II {BETHESDA II}). After consultation with the endocrinologist, total thyroidectomy was planned to address persistent hyperthyroidism. Prior to surgery, repeat thyroid function tests (TFTs) showed a persistently suppressed TSH level (0.013 mIU/L), with free T3 and free T4 within normal limits (Table 2). Preoperatively she remained inpatient to minimize the risk of thyroid storm and was maintained on neomercazole 15 µg daily once. The patient underwent total thyroidectomy (Figures 2, 3).

**Table 2:** Preoperative thyroid function test (TFT) values.

Laboratory investigations	Patient's value	Institutional value
Thyroid-stimulating hormone	0.013 mIU/L	0.5-8.9 mIU/L
Free T3	2.45 ng/dL	2.1-4.4 ng/dL
Free T4	1.19 ng/dL	0.8-2.7 ng/dL

**Figure 4:**
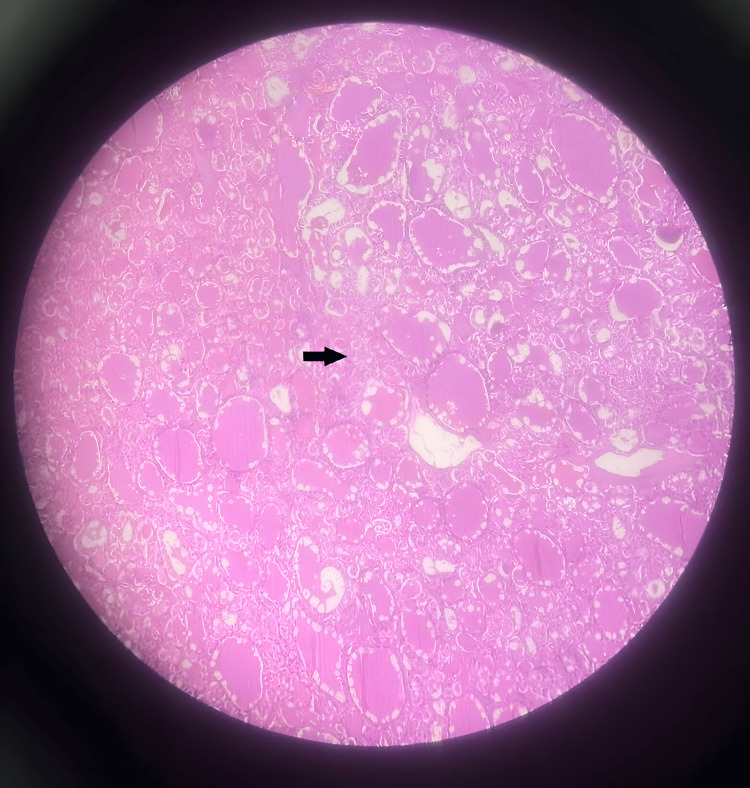
Tumor composed predominantly of Hürthle cells (arrow) (H&E, 10x).

**Figure 5:**
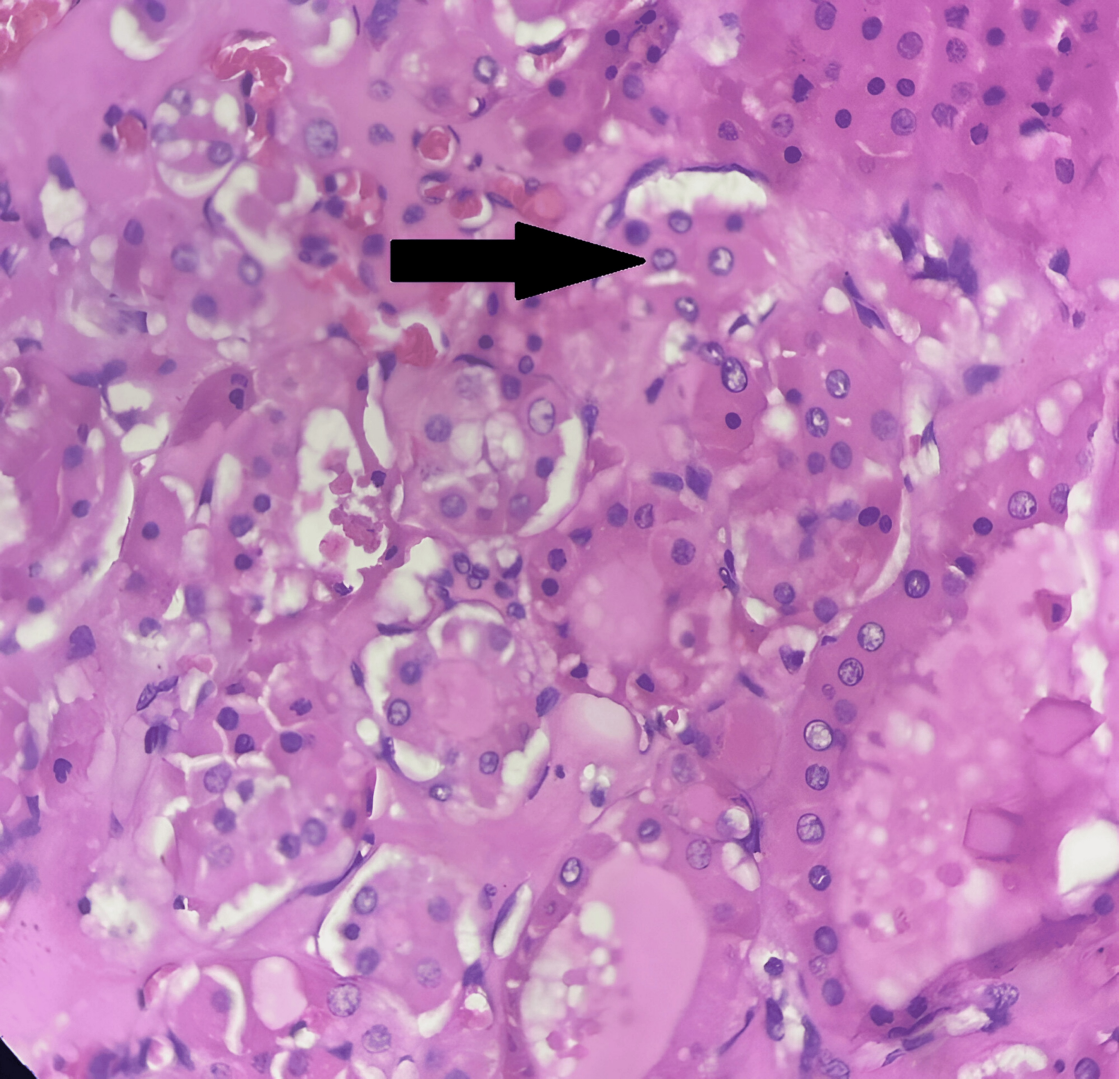
Tumor composed predominantly of Hürthle cells shown in high power magnification (arrow) (H&E).

**Figure 6:**
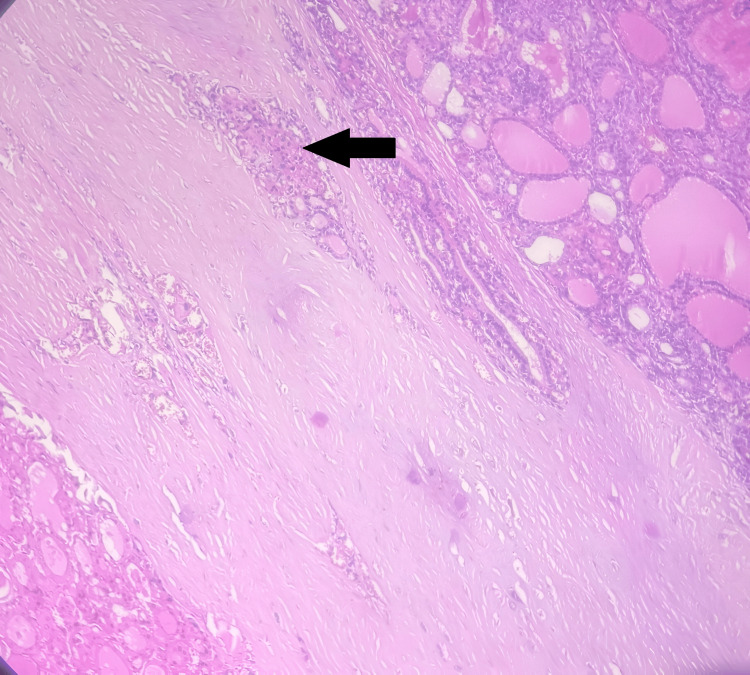
Tumor islands in capsule with questionable invasion (arrow).

The postoperative period was uneventful. Follow-up Iodine-131 (I^131^) whole body scan revealed no evident residual thyroid. Histopathological examination revealed a low-risk follicular cell-derived thyroid neoplasm - thyroid follicular tumor of uncertain malignant potential (the tumor is composed of predominantly Hürthle cells >75% with foci of endocrine atypia with questionable capsular invasion) (Figures 4-6). Mitotic rate <3 mitosis/2 mm^2^. All margins were negative for carcinoma.

Postoperatively, hematological parameters are mentioned in Table 3. The patient was started on tab. thyroxine 100 µg once daily. The patient experienced no complications on follow-up for six months, with regular TFT monitoring.

**Table 3:** Postoperative thyroid function test (TFT) values.

Laboratory investigations	Patient's value	Institutional value
Thyroid-stimulating hormone	87.9 mIU/L	0.5-8.9 mIU/L
Free T4	0.25 ng/dL	0.8-2.7 ng/dL
Ionized calcium	8.5 mg/dL	8.5-10.2 mg/dL

## Discussion

Thyroidectomy is one of the definitive treatment options for hyperthyroidism, particularly in cases where other therapies, such as antithyroid medications or radioactive iodine, are ineffective, contraindicated, or not preferred. It is often considered for patients with large goiters causing compressive symptoms, suspicion or presence of thyroid cancer, coexisting nodules, or in women planning pregnancy, where rapid control of thyroid function is needed. Thyroidectomy offers rapid and definitive resolution of hyperthyroidism, eliminating the need for lifelong antithyroid medication. However, it carries surgical risks, including bleeding, infection, recurrent laryngeal nerve injury (which may affect voice), and hypoparathyroidism due to parathyroid gland damage. Postoperatively, patients typically require lifelong thyroid hormone replacement therapy.

In India, there is a high prevalence of benign thyroid nodules - both toxic and nontoxic - due to endemic iodine deficiency. However, the incidence of thyroid malignancy among patients with hyperthyroidism remains low.

Follicular carcinoma of the thyroid typically presents in a euthyroid state and is often identified as a cold nodule on thyroid scans. Its association with hyperthyroidism is rare and of uncertain significance, with reported incidence rates ranging from 0.3% to 16.6% [8].

Genetic research on cancer development in hyperthyroid patients suggests that TSH is a significant contributor. In cases of thyroid gland failure, sustained and intense stimulation by TSH has been observed to elevate cyclic adenosine monophosphate (cAMP) levels, leading to increased growth and proliferation of follicular cells. This same stimulatory effect of TSH has also been noted in both benign (adenomatous) and malignant (carcinomatous) thyroid tissues.

An elevated TSH level, which can be caused by iodine deficiency, a reduced ability to produce thyroid hormone, or the consumption of goitrogens (substances that interfere with thyroid function found in diet or medication), is linked to an increased risk of thyroid cancer [9].

Hürthle cell carcinomas (HCC) are relatively rare in regions with sufficient iodine intake. Despite the rarity of HCC, patients showed relatively good outcomes, with persistent or recurrent disease occurring in 8.2% of patients, and no deaths attributed to HCC during a median follow-up of 8.5 years. It is characterized by the presence of Hürthle cells or oxyphilic cells, derived from the follicular epithelium of the thyroid [10]. Compared to other forms of well-differentiated thyroid cancers, Hürthle cell carcinoma (HCC) is associated with higher rates of recurrence and mortality [11]. Distant metastases occur in approximately 15-34% of cases [12]. Several features help differentiate benign from malignant Hürthle cell carcinomas (HCC). Tumor size is a key distinguishing factor, with HCCs typically being significantly larger (mean size: 4.0 cm) than adenomas (mean size: 2.4 cm). However, factors such as patient age, sex, history of neck irradiation, tumor bilaterality, and the presence of concurrent thyroid cancers do not reliably differentiate between benign and malignant Hürthle cell neoplasms [13]. Tumor size greater than 4 cm, vascular invasion involving more than four foci, evidence of mitosis, solid or trabecular growth patterns, extrathyroidal extension, and lymph node metastases are histologic features associated with an increased risk of recurrence. Notably, extensive vascular invasion has been strongly linked to a shorter five-year disease-free interval and poorer disease-specific survival outcomes [14]. Prognosis is heavily influenced by the extent of vascular invasion, with a greater number of affected vessels correlating with a significantly poorer outlook, as mortality exceeds 90% at 10 years. Additional prognostic factors include age, sex, cancer stage, and the extent of disease spread. Poor prognostic indicators include older age, male sex, larger tumor size at the time of diagnosis, extrathyroidal extension, and advanced stage at presentation [15].

## Conclusions

This report demonstrates that total thyroidectomy can be a safe and highly effective treatment for refractory hyperthyroidism, offering a definitive resolution when medical and radioiodine therapies are not suitable or have proven unsuccessful. The patient in this instance experienced a significant improvement in their clinical status and achieved stable euthyroidism postoperatively without major complications. While thyroidectomy is associated with a risk of permanent hypothyroidism requiring lifelong levothyroxine replacement, in cases of severe, persistent hyperthyroidism, particularly when other treatment options are limited, it provides a valuable and potentially life-changing intervention. The presence of hyperthyroidism should not exclude the possibility of thyroid cancer. Clinicians should be aware that thyroid cancer and hyperthyroidism can coexist, and this possibility must be considered in patient management.
